# Prevalence of Neurovascular Microemboli After Transcatheter Aortic Valve Replacement

**DOI:** 10.1016/j.jscai.2023.101180

**Published:** 2023-11-30

**Authors:** Navneet Sharma, Ryan F. Heslin, Saadat U. Aleem, John Medamana, Leyla Gasimli-Gamache, Jeanwoo Yoo, Varun Bhasin, Peter J. Avvento, Jose Wiley, Thomas V. Billfinger, Henry J. Tannous, Puja B. Parikh, Smadar Kort, Nicos Labropoulos, George D. Dangas, John P. Reilly, Robert T. Pyo

**Affiliations:** a Division of Cardiology, Stony Brook University Medical Center, Stony Brook, New York; b Department of Medicine, Stony Brook University Medical Center, Stony Brook, New York; c Divison of Cardiology, Tulane University, New Orleans, Louisiana; d Division of Cardiothoracic Surgery, Stony Brook University Medical Center, Stony Brook, New York; e Division of Vascular Surgery, Stony Brook University Medical Center, Stony Brook, New York; f Division of Cardiology, The Mount Sinai Hospital, New York, New York

**Keywords:** distal embolic protection, high intensity transient signals, stroke, transcatheter aortic valve implantation, transcranial Doppler

## Abstract

**Background:**

Neurolotic sequelae after transcatheter aortic valve replacement (TAVR) can cause significant morbidity and mortality. Transcranial Doppler (TCD) imaging can show real-time high intensity transient signals (HITS), which reflect active microembolization. Although it is well known that intraprocedural microembolism occurs, it is not known if this embolic phenomenon continues in the postprocedural period. We investigated whether microemboli occur post-TAVR and whether we could determine any clinical, procedural, or echocardiographic predictors.

**Methods:**

We evaluated HITS in 51 consecutive patients undergoing unprotected TAVR with low-, intermediate-, or high-risk Society of Thoracic Surgeons score. Patients were excluded if they did not have temporal windows for insonation of the middle cerebral artery or if they were not willing to participate. Primary outcomes of HITS 24 hours post-TAVR were observed using a Philips iU22 TCD. TCD was performed at 3 time points (pre-, peri-, and post-TAVR) for each patient, before, during, and 24 hours postprocedure.

**Results:**

While no HITS were detected in any of the patients preoperatively, all patients had HITS during the procedure. Interestingly, 56.8% had HITS 24 hours post-TAVR. One patient with HITS post-TAVR had a stroke 48 hours after TAVR.

**Conclusion:**

We observed a high prevalence of microemboli 24 hours post-TAVR. None of the predictors for intraprocedural microembolism seemed to play an important role for post-TAVR microemboli.

## Highlights

• A high incidence of late high intensity transient signals was noted.
• Late signals showed no association with published variables.
• Late embolism may be why embolic protection is ineffective.
• Further research is required to correlate late embolism with clinical events.

## Central Illustration

**Figure:**
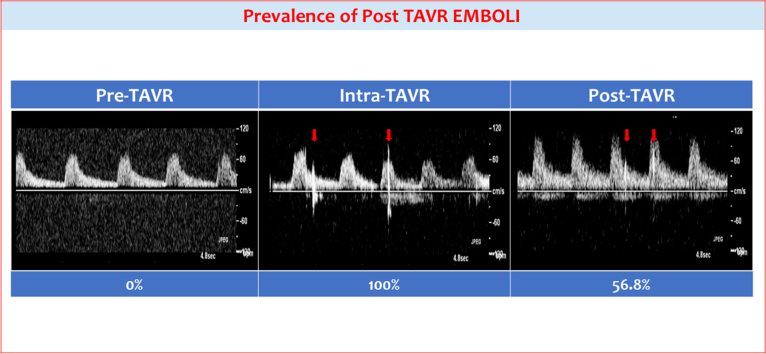

## Introduction

The volume of transcatheter aortic valve replacements (TAVR) has rapidly expanded and includes patients with lower surgical risk profiles. Since 2002, there have been 200,000 TAVR performed worldwide, and more than 40,000 procedures were done in the United States from 2012 to 2014. As of 2022, approximately 1.5 million patients have received TAVR.^1^, ^2^, ^3^, ^4^ It is now more important than ever to minimize complications.

A feared and debilitating adverse outcome of TAVR is a cerebrovascular event (CVE), which can range from neurocognitive dysfunction to transient ischemic attack and stroke.^5^ The manipulation of the aorta and stenotic valve during TAVR contributes to an inherent CVE risk comparable to that associated with surgical replacement.^6^ In fact, some studies suggest nearly twice the number of strokes at 30 days and 1 year for TAVR compared to surgical replacement.^7^^,^^8^ Although the randomized controlled TAVR trials estimate the post-TAVR stroke rate to be 2% to 5%,^9^^,^^10^ more recent real world data suggests this may actually be higher.^11^, ^12^, ^13^, ^14^

TAVI causes CVE through emboli that can be detected during the procedure by using transcranial Doppler (TCD).^15^ Emboli are detected by TCD as high-intensity transient signals (HITS), which correlate with periods of increased valvular manipulation and deployment.^5^^,^^16^, ^17^, ^18^, ^19^ Of note, CVE do not occur immediately after the procedure. Indeed, 85% of strokes happened in the first 7 days, with the highest reported risk at 24 to 48 hours post-TAVR.^5^^,^^9^ Moreover, use of distal embolic protection devices (DEPD) have not shown significant reduction in ischemic brain lesions (IBL) or clinical outcomes post-TAVR.^12^^,^^13^^,^^20^^,^^21^ In contrast to the TAVR literature, there are both clinical and ultrasound data for patients undergoing carotid revascularization procedures that demonstrate a relationship between vulnerable plaque (as measured by HITS) and incidence of stroke.^22^, ^23^, ^24^, ^25^ Therefore, major concerns remain regarding potential ongoing risk of microemboli in the post-TAVR period when DEPD would have been removed. Despite this, there is complete lack of data on microemboli in the post-TAVR period.

The primary objective of our pilot study was to utilize TCD to determine the frequency of microemboli, observed as HITS, in the post-TAVR period. We sought to identify clinical, procedural, and echocardiographic parameters that may predict the risk of microemboli during this period.

## Methods

Institutional review board approval was obtained at Stony Brook University Hospital. We included patients undergoing TAVR with low, intermediate, or high risk as determined by Society of Thoracic Surgeons score. Patients were excluded if they had temporal windows unsuitable for insonation of the middle cerebral artery (MCA) or if they were not willing to participate. The primary outcome measured was a categorical determination if HITS occurred in the 24-hour period post-TAVR. All scans—pre, peri, and post—were 30 minutes in duration.

**Figure 1:**
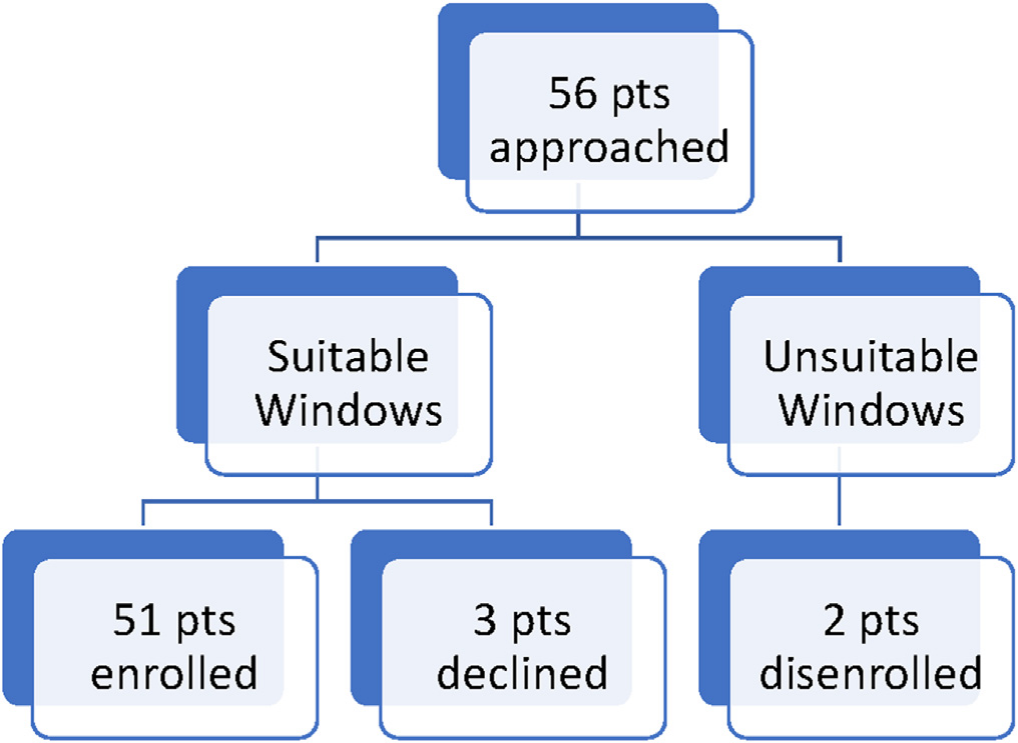
**Patient flow diagram: 56 patients (pts) were studied.** Two patients were excluded because of poor transcranial Doppler windows. Three patients with good windows withdrew consent. Fifty-one patients were included in the final analysis.

Written consent was obtained from the participating subjects. We prospectively studied 56 consecutive subjects undergoing unprotected TAVR, age ≥18 years. Fifty-one were included in the final analysis, as 3 withdrew consent, and 2 had suboptimal TCD image quality. Clinical and laboratory data including past medical history, demographics, and medications were obtained from subject interview and chart review (Figure 1).

### TAVR

We enrolled 51 consecutive patients undergoing TAVR. Patients eligible for TAVR had severe symptomatic aortic stenosis on echocardiography (valve area <1 cm^2^, peak velocity >4 m/s, mean pressure gradient >40 mm Hg). All patients were evaluated by the Stony Brook University Hospital TAVR committee. Patients were deemed eligible for TAVR after consensus from the interventional cardiologist and cardiothoracic surgery.

### TCD and HITS

**Figure 2:**
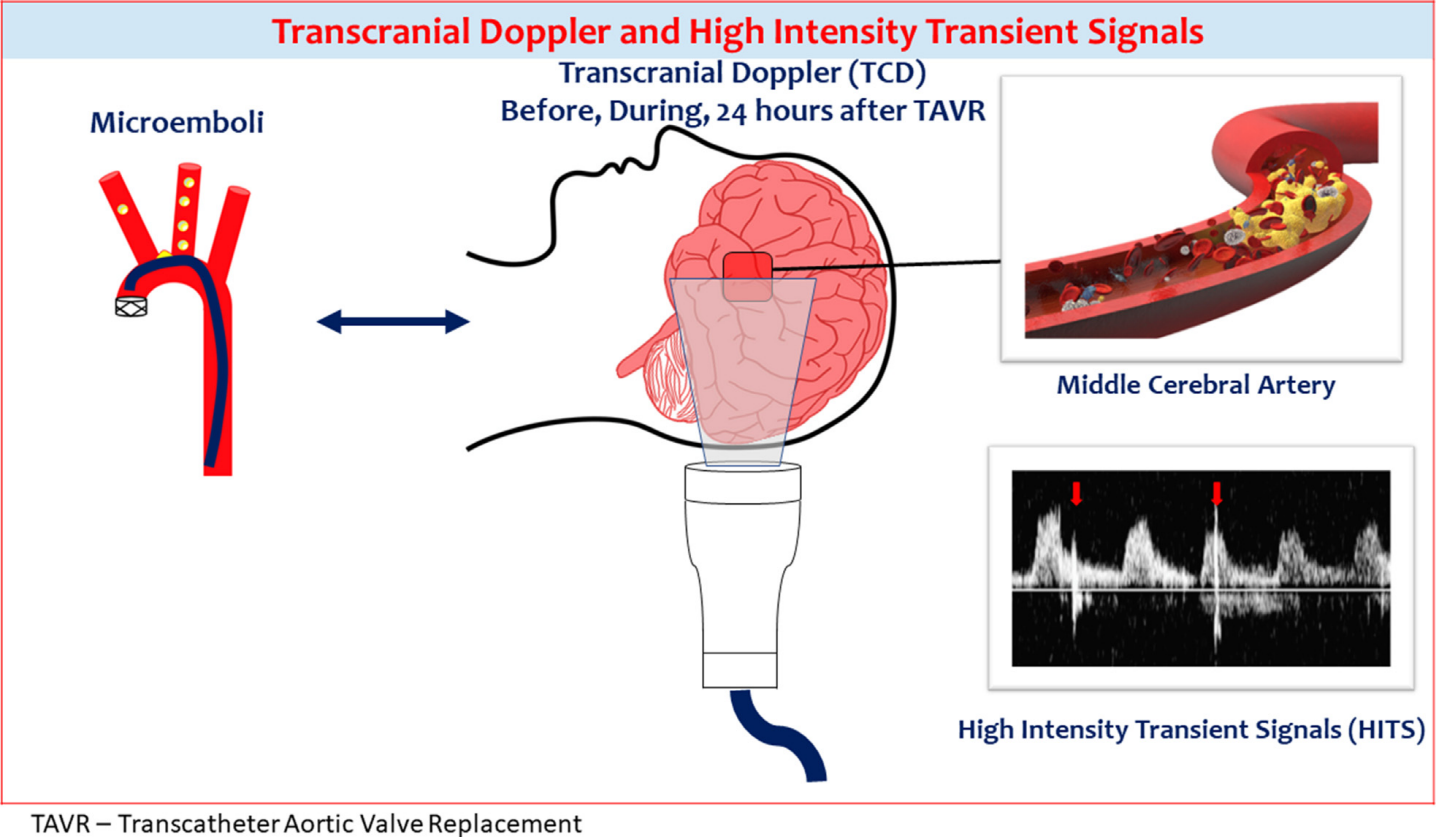
**Schematic representation of transcranial Doppler (TCD): middle cerebral artery was insonated using Philips iU22.** Each patient was scanned at 3 different time points: before, during, and 24 hours after TAVR. HITS were defined as unidirectional transient signals (duration <300 ms) that have an amplitude >3 decibels higher than the background blood flow in the Doppler velocity spectrum. HITS are highlighted with red arrows. HITS, high-intensity transient signals; TAVR, transcatheter aortic valve replacement.

Transcranial Doppler was performed at 3 time points for each patient: pre-, peri-, and 24 hours post-TAVR. The entire TCD was performed on the left MCA using a Philips iU22 (Figure 2). The recorded TCD examinations were reviewed for the presence of HITS, indicative of embolic events in the MCA, by 3 investigators performing the scans. These interpretations were verified by a field expert (N.L.).^17^^,^^22^^,^^23^ HITS were defined as transient signals (duration <300 ms) that have an amplitude >3 decibels higher than the background blood flow and are unidirectional in the Doppler velocity spectrum. These criteria for HITS have been previously described in Doppler guidelines.^24^

### Echocardiography

Two-dimensional transthoracic echocardiography was performed on all patients per the routine pre-TAVR evaluation using Philips or General Electric systems. All patients also had post-TAVR echocardiography prior to discharge. All studies were interpreted using Syngo Dynamics software. Measurements and calculations were completed based upon institutional protocols, adapted from recommendations from the American Society for Echocardiography. Anatomic and hemodynamic data collected from transthoracic echocardiograms pre-TAVR and 24 hours post included: left ventricular ejection fraction, aortic valve area, transaortic valve mean pressure gradients, dimensionless index, peak aortic jet velocity, stroke volume index, E wave/A wave ratio, peak E wave velocity/peak e' wave velocity ratio, left atrial volume index (LAVi), pulmonary artery systolic pressure, tricuspid annular plane systolic excursion, Zva ([systolic blood pressure + mean gradient]/stroke volume index), and peak tricuspid regurgitation velocity.

### Computed tomography

All patients underwent contrast-enhanced, electrocardiogram-gated cardiac computed tomography (CT) and helically-acquired CT angiogram of the chest, abdomen, and pelvis per the routine pre-TAVR workup. Maximum intensity projection, 3-dimensional, and multiplanar reconstructions were performed. Total aortic valve calcium score, ascending aortic diameter to catheter diameter ratio (at the level of the pulmonary artery), aortic root angle, systolic annular perimeter, and area were collected using 3Mensio Structural Heart software (Pie Medical Imaging). The aortic arch was not imaged in all patients and therefore was excluded from the analysis.

### Statistical analysis

Continuous data were analyzed using the *t* test. All values were expressed as mean ± SD. The continuous variables were further analyzed using nonparametric testing. The Wilcoxon rank sum analysis did not show discrepancy in statistical significance of any variables when compared to *t* test analysis of the continuous variables (Supplemental Data). Univariate associations between study variables were analyzed using Fisher exact analysis for categorical variables. Multivariate linear regression was used to determine independent correlates of HITS, using all variables with P < .1 on univariate analysis. All statistical analyses were performed with Stata Version 13 software (StataCorp LP). A 2-sided *P* value <.05 was considered to be statistically significant.

## Results

Overall, 51 consecutive patients underwent TCD evaluation at 3 distinct time points (pre-, peri-, and post-unprotected TAVR (transfemoral n = 49, transaxillary n = 2). The average age was 78.3 ± 9.9 years and average Society of Thoracic Surgeons score was 5.4% ± 4.2%. There were 39 males and 12 females in the cohort. Among them, 46 patients had coronary artery disease, 21 were on P2Y12 inhibitor, and 15 were on anticoagulation. Pre-existing atrial fibrillation was present in 15.7% of patients during the study period. None of the patients developed new-onset atrial fibrillation within 30 days of TAVR. Average aortic valve area was 0.74 ± 0.21 cm^2^, mean pressure gradient 42.5 ± 15.9 mm Hg, peak velocity 4.09 ± 0.74 m/s, and left ventricular ejection fraction 56.5% ± 11.2%. Edwards SAPIEN valves were placed in 37 patients and Medtronic valves in 14. Baseline characteristics for the cohort are summarized in Table 1. None of the patients demonstrated pre-TAVR HITS, but all had HITS during the procedure (3 patients did not have intraprocedural TCD, 2 due to surgical cut downs and 1 due to oxygen requirements during the case that limited windows). Post-TAVR HITS were detected in 29 patients, representing 56% of the cohort (Central Illustration). Of the 51 patients, there was 1 clinically confirmed stroke at 48 hours post-TAVR; this patient had HITS 24 hours postprocedure.

**Table 1:** Baseline characteristics.

Characteristics	Value
Age, y	78.3 ± 10.0
Male sex	39 (76.5)
Body mass index, kg/m^2^	28.4 ± 6.1
Society of Thoracic Surgeons score	5.4 ± 4.3
Coronary artery disease	46 (90.2)
Prior cerebrovascular accident	5 (9.8)
Creatinine, mg/dL	1.7 ± 1.8
Glomerular filtration rate, mL/min/1.73 m^2^	56.2 ± 25.0
Hemoglobin A1c, %	6.0 ± 1.3
Atrial fibrillation	8 (15.7)
Anticoagulation	15 (29.4)
Aortic valve area, cm^2^	0.7 ± 0.2
Mean pressure gradient, mm Hg	42.5 ± 15.9
Left ventricular ejection fraction, %	56.5 ± 11.2
Systolic annular perimeter, mm	80.9 ± 8.1
Systolic annular area, mm^2^	498.4 ± 97.9

Values are presented as n (%) or mean ± SD.

**Central Illustration:**
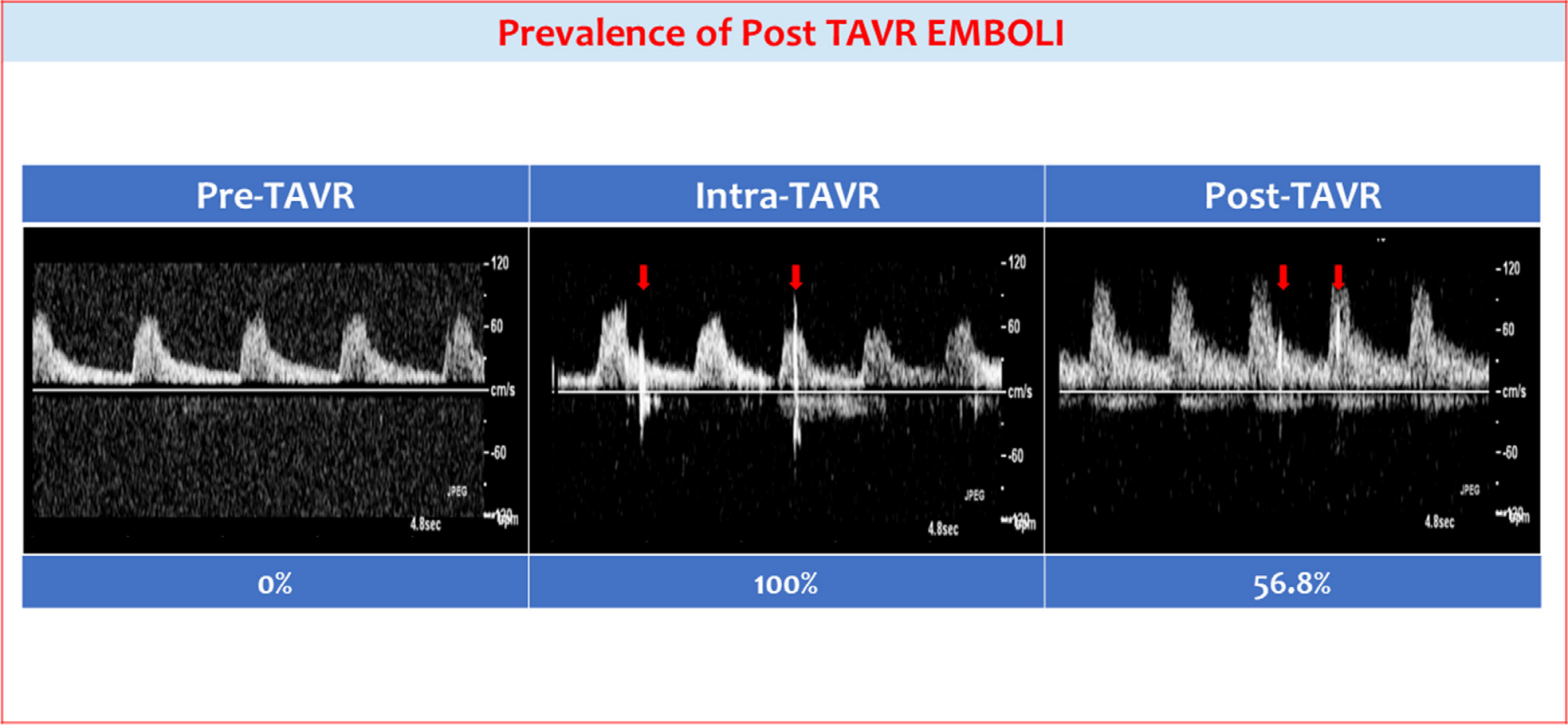
No patients had pre-transcatheter aortic valve replacement high-intensity transient signals (HITS). All patients with intra-TAVR transcranial Doppler had evidence of HITS. Of the patients, 56.8% continued to have HITS at 24 hours post-TAVR.

Univariate analysis for post-HITS according to patients’ characteristics (Table 2), procedural characteristics (Table 3), medication use (Table 4), echocardiographic parameters (Table 5), and CT characteristics (Table 6) are summarized in respective tables. Univariate analysis showed significant association of post-TAVR HITS with absolute change in LAVi (*P* = .006) (Table 5). Additionally, pre-TAVR LAVi also significantly predicted post-TAVR HITS. A larger ratio of ascending aorta length to diameter ratio in relation to the sheath size corrected by body surface area showed a trend toward developing HITS (*P* = .07) (Table 6). Multivariate analysis revealed absolute change in LAVi remained an independent predictor for reducing microemboli postprocedure (adjusted odds ratio, 0.86; 95% CI, 0.74-0.97, *P* =.01) (Table 7).

**Table 2:** Patient characteristics.

Variable	HITS (n=29)	No HITS (n=22)
Age	79.3 ± 9.9	76.9 ± 10.1
Male sex	20 (69)	19 (27)
Coronary artery disease	25 (86)	21 (95)
Hypertension	27 (93)	18 (82)
Hyperlipidemia	27 (93)	18 (82)
Smoking	18 (62)	11 (50)
Prior cerebrovascular accident	3 (10)	2 (9)
Atrial Fibrillation	4 (14)	4 (18)
Prior coronary artery bypass grafting	5 (17)	3 (14)
Ascending aortic calcification	23 (79)	15 (68)
Society of Thoracic Surgeons score	6.0 ± 5.2	4.6 ± 2.6
Hemoglobin, g/dL	12.2 ± 1.7	11.9 ± 2.5
Hematocrit, %	38.1 ± 5.1	37.3 ± 7.4
International normalized ratio	1.1 ± 0.1	1.2 ± 0.3
Creatinine, mg/dL	2.0 ± 2.2	1.5 ± 1.5
Glomerular filtration rate	58.7 ± 24.0	53.0 ± 26.6
Body mass index	27.9 ± 6.4	29.0 ± 5.7
Body surface area, m^2^	1.9 ± 0.3	2.0 ± 0.2
Hemoglobin A1c, %	6.0 ± 1.4	6.0 ± 1.1
Coronary arteries		
Left Main		
No Disease (0% Stenosis)	22 (76)	14 (64)
Mild Disease (10-30% Stenosis)	3 (10)	6 (27)
Moderate Diseases (30-60% Stenosis)	3 (10)	0
Severe Disease (>60% Stenosis)	2 (6.9)	1 (4.5)
Left Anterior Descending		
No Disease (0% Stenosis)	7 (24)	7 (32)
Mild Disease (10-30% Stenosis)	6 (21)	4 (18)
Moderate Disease (30-70% Stenosis)	6 (21)	4 (18)
Severe Disease (>70% Stenosis)	10 (34)	7 (32)
Left Circumflex		
No Disease (0% Stenosis)	15 (52)	10 (45)
Mild Disease (10-30% Stenosis)	3 (10)	5 (2.3)
Moderate Disease (30-70% Stenosis)	5 (17)	2 (9.0)
Severe Disease (>70% Stenosis)	6 (21)	5 (23)
Right Coronary Artery		
No Disease (0% Stenosis)	10 (34)	8 (36)
Mild Disease (10-30% Stenosis)	4 (14)	5 (23)
Moderate Disease (30-70% Stenosis)	7 (24)	2 (9.0)
Severe Disease (>70% Stenosis)	8 (28)	7 (32)
Left Internal Coronary Artery		
No Disease (0% Stenosis)	26 (90)	19 (86)
Mild Disease (30-50% Stenosis)	0	2 (9.0)
Moderate Disease (50-70% Stenosis)	2 (6.9)	1 (4.5)
Severe Disease (>70% Stenosis)	1 (3.4)	0

Values are presented as n or mean ± SD.

HITS, high intensity transient signals.

**Table 3:** Procedural characteristics.

Variable	HITS (n = 29)	No HITS (n = 22)	*P*
Valve type			
SAPIEN (Edwards)	20	17	0.51
Medtronic	9	5	
Valve size			
23 mm	5	4	1.00
26 mm	11	9	
29 mm	13	9	
Predilatation			
Performed	9	9	0.47
Not performed	20	13	
Predilatation size			
20 mm	3	3	0.80
22 mm	0	1	
23 mm	4	3	
24 mm	1	0	
25 mm	1	2	
None	20	13	
Postdilation			
Performed	1	3	0.30
Not performed	28	19	
Postdilation size			
23 mm	1	2	0.38
25 mm	0	1	
None	28	19	
Valve in valve			
Yes	1	2	0.57
No	28	20	
Fluoroscopy time, min	21.2 ± 11.1	18.7 ± 6.8	0.35
Sheath size, mm	4.9 ± 0.3	5.01 ± 0.31	0.16

Values are presented as n or mean ± SD.

HITS, high-intensity transient signals.

**Table 4:** Medications.

Variable	HITS (n = 29)	No HITS (n = 22)	*P*
Aspirin	22 (76)	18 (82)	0.74
Thienopyridines	12 (41)	9 (41)	0.97
Beta blocker	18 (62)	11 (50)	0.39
Angiotensin-converting enzyme inhibitor	14 (48)	8 (36)	0.40
Mineralocorticoid receptor antagonist	2 (7)	1 (5)	1.00
Statin	22 (76)	17 (77)	0.91
Anticoagulation	10 (34)	5 (23)	0.36

Values are presented as n (%).

HITS, high intensity transient signals.

**Table 5:** Echo parameters.

Variable	Pre-TAVR			Δ Post-TAVR		
	HITS (n = 29)	No HITS (n = 22)	*P*	HITS	No HITS	*P*
Ejection fraction, %	58.1 ± 1.9	54.5 ± 2.6	0.26			
Aortic valve area, cm^2^	0.7 ± 0.2	0.8 ± 0.2	0.32			
Aortic valve area index	0.4 ± 0.1	0.4 ± 0.1	0.91			
Dimensionless obstructive index	0.2 ± 0.1	0.2 ± 0.06	0.75	0.3 ± 0.1	0.2 ±.01	0.50
Mitral pressure gradient, mm Hg	40.1 ± 13.3	45.7 ± 18.7	0.213	29.5 ± 12.4	33.6 ± 17.3	0.33
Pressure volume, m/s	4.0 ± 0.7	4.2 ± 0.8	0.268	1.3 ± 0.7	1.4 ± 0.8	0.84
Systolic blood pressure, mm Hg	131.1 ± 21.5	140.3 ± 23.3	0.15	23.6 ± 18.9^a^	23.1 ± 14.1^a^	0.93
Stroke volume index	33.4 ± 8.4	35.0 ± 11.9	0.57	0.6 ± 1.5	0.4 ± 0.5	0.45
E/e'	17.0 ± 7.9	18.0 ± 7.9	0.68	3.7 ± 3.4^a^	5.4 ± 4.3^a^	0.12
Septal E’	3.3 ± 2.9	4.5 ± 2.1	0.10	2.6 ± 2.1^a^	1.8 ± 1.9^a^	0.15
Lateral E’	4.6 ± 4.0	5.4 ± 2.7	0.43	3.7 ± 3.4^a^	2.1 ± 1.9^a^	0.04
TR Vmax	2.6 ± 0.5	2.5 ± 0.6	0.29	0.4 ± 0.3^a^	0.5 ± 0.5^a^	0.48
E/A	1.2 ± 1.4	1.0 ± 0.8	0.66	0.6 ± 1.5^a^	0.4 ± 0.4^a^	0.44
Left atrial volume index	34.9 ± 11.8	43.1 ± 16.5	0.04	5.2 ± 5.4^a^	9.9 ± 6.4^a^	0.01
Ventriculo-arterial impedance, mm Hg mL^−1^ m^−2^	6.0 ± 2.3	5.4 ± 1.5	0.33	1.1 ± 2.1	1.9 ± 2.3	0.19

Values are expressed as mean ± SD.

Δ, change in parameter; E, peak E wave velocity; E/A, E wave/A wave; E’, peak E' wave velocity; TAVR, transcatheter aortic valve replacement; TR Vmax, maximal tricuspid regurgitation velocity.

a Absolute value.

**Table 6:** Computed tomography parameters.

Variable	HITS (n = 29)	No HITS (n = 22)	*P*
Root angle, °	49.9 ± 10.1	51.1 ± 8.2	0.64
Ascending aorta diameter, cm	3.5 ± 0.4	3.5 ± 0.3	0.42
Ascending aorta calcium	23 (79)	15 (68)	0.37
Systolic annular perimeter, mm	80.3 ± 7.1	81.7 ± 9.4	0.55
Systolic annular area, mm^2^	489.7 ± 84.6	510.0 ± 114.2	0.47
Aorta ascending height, cm	9.7 ± 0.9	9.6 ± 0.9	0.91
LDRS by BSA	0.74 ± 0.14	0.68 ± 0.11	0.07

Values are presented as n (%) or mean ± SD.

BSA, body surface area; HITS, high intensity transient signals; LDRS, aortic length diameter ratio in relation to sheath size.

**Table 7:** Multivariate analysis.

Variable	Adjusted odds ratio	95% CI	*P*
Δ Left atrial volume index^a^	0.86	0.74 – 0.97	0.01
Δ Lateral E′^a^	1.33	0.93 – 1.90	0.12
LDRS by BSA	92.95	0.32 – 2.7×10^4^	0.08
Preseptal E’ (peak E' wave velocity)	1.06	0.74 – 1.52	0.18

Δ, change in parameter; BSA, body surface area; LDRS, aortic length diameter ratio in relation to sheath size.

a Absolute value.

## Discussion

HITS detected by TCD during cardiovascular procedures imply embolic phenomenon. HITS result from atherosclerotic emboli during percutaneous carotid intervention.^23^, ^24^, ^25^ Similarly, intraprocedural HITS during TAVR are known to be associated with embolization of solid material such as thrombus, calcium, and other valvular and vascular architecture that are captured in distal embolic protection devices.^26^^,^^27^ Diffusion-weighted magnetic resonance imaging in patients undergoing TAVR without DEPD have shown increased IBL at 4 ± 2 days that were associated with a decline in Montreal Cognitive Assessment or National Institute of Health Stroke Scale in 59.4% of patients.^14^ These observations imply that embolism may be necessary in the pathogenesis of CVE in patients undergoing TAVR and that HITS detected with TCD are an efficacious way to detect this event.

While prior studies have demonstrated the presence of HITS during TAVR, our study is the first to examine the occurrence of HITS beyond the immediate periprocedural period in patients undergoing TAVR. Over 56.8% of the patients studied demonstrated HITS 24 hours post-TAVR. None of the patients demonstrated HITS pre-TAVR. All of our patients demonstrated HITS peri-TAVR, and this observation is consistent with previously reported rates of 70% to 100% during unprotected TAVR.^12^^,^^13^^,^^21^

Intraprocedural embolic events do not explain all cases of CVE in patients undergoing TAVR. The PARTNER study showed that the risk of CVE persisted after the procedure and peaked at 48 hours. CVE risk fell to 0.8% per year by 2 weeks.^9^ Additionally, large registry data have shown an average delay of 48 hours between TAVR and clinical manifestation of stroke.^10^ These findings suggest that an active phenomenon that occurs beyond the intraprocedural period may contribute to CVE in patients undergoing TAVR. Our study identifies HITS that extend beyond the intraprocedural period in almost 60% of our patients. Our findings suggest that microembolization continues postprocedurally, as far out as 24 hours postprocedure. This late embolic phenomenon may contribute to CVE in these patients, and more precisely, suggests a mechanism for the delayed clinical manifestations of CVE in patients undergoing TAVR.

In the SENTINEL trial, patients treated with a dual-filter DEPD demonstrated increasing IBL burden between days 2 and 7.^12^ Additionally, despite the use of DEPD that covered all 3 major aortic arch branches, 73% to 95% of patients have been observed to have IBL 2 to 5 days post-TAVR.^13^^,^^20^ Furthermore, the use of DEPD is not associated with a significant reduction in IBL at 7 days post-TAVR. Moreover, the use of DEPD has not been shown to reduce CVE in randomized controlled trials and large registries.^21^ Postprocedural embolic events, as implied by our data, could occur when DEPD are removed. These late events may explain why DEPD do not confer complete protection from microemboli in patients undergoing TAVR and may play an important role in the development of CVE.

The overall stroke rate in our cohort was 2.0%, which is consistent with previous reports.^28^, ^29^, ^30^, ^31^, ^32^, ^33^ One of our patients developed a stroke 48 hours after the procedure, and HITS were detected at the 24-hour mark. Notably, the patient was awake and alert and demonstrated no neurologic deficits up to 12 hours after TAVR. Other patients with HITS measured at a single time point at the time of TCD did not manifest clinical CVE. A single time point underrepresents the total burden of microemboli, and it is likely that there were continuous, multiple embolic events over a 24-hour period in patients who demonstrated post-TAVR HITS. The present study treated microemboli as a categorical variable, and as such, the total volume of HITS was not evaluated as a continuous variable. We hypothesize that the impact of HITS on the clinical manifestation of CVE likely depends on the total volume of microemboli and cerebral vascular reserve.

We hypothesize that the mechanism of post-TAVR HITS may be related to aortic injury. It is conceivable that manipulation of larger equipment in smaller aortas may increase the risk of aortic injury and HITS. Interestingly, in our study, a larger ascending aortic length to diameter ratio in relation to sheath size adjusted for body surface area was associated with a trend toward predicting HITS. Varying degrees of injury to the aorta and aorta/valve complex can occur during TAVR and may cause the formation of mural thrombus.^34^^,^^35^ It is possible that subclinical injury to the aorta sustained during delivery of TAVR equipment coupled with conditions favorable for thrombi formation such as atherosclerosis may contribute to late embolic events that were detected by TCD in our study.

In our study, periprocedural variables associated with immediate embolic events such as predilation, valve type, and valve-in-valve procedures showed no significant association with late post-TAVR HITS. Furthermore, patient characteristics such as atrial fibrillation, diabetes, use of anticoagulants or aspirin, and prior history of stroke were not associated with late post-TAVR HITS. Finally, anatomic characteristics such as presence of ascending aorta calcification, annular area, and root angle were not associated with late HITS in our study. Interestingly, many of these characteristics are associated with embolic events in published studies. However, none of these studies evaluated late HITS. The absolute change in LAVi was inversely related to the chance of late HITS in our study. The clinical relevance of this finding is uncertain. Furthermore, additional research is required to determine if patients who had unsuitable acoustic windows for TCD have systemic differences that may impact their risk of CVE.

It has been shown that HITS occur during wire manipulation, device advancement, and valve deployment. These maneuvers occur during the procedure and are unlikely to cause late HITS. Although the native valve may be the source of emboli during the procedure, it is not likely the source of continued emboli after TAVR valve deployment because the native valve leaflets are excluded behind the valve struts. No characteristic of the native valve was associated with late HITS. Our findings leave open the possibility that the formation of mural thrombus on the aorta may play a role in delayed embolization. Successfully identifying baseline characteristics that predict late microemboli, as demonstrated by HITS, may provide more accurate risk assessment for CVE due to TAVR.

There are several study limitations: (1) no data were available on aortic arch type and this may have impacted our analysis to detect patients that are more prone to have HITS; (2) this was a pilot study and as such was not powered for CVE outcomes; (3) diffusion-weighted magnetic resonance imaging was not performed to define IBL and correlate with HITS, which may be the focus of future studies; and (4) post-TAVR HITS was a categorical outcome in our study. HITS were assessed at a single time point. Further studies employing continuous monitoring are needed to assess the impact of density (HITS per unit time) and total volume of HITS on outcomes.
