# Supplementary material for: Prevalence of Neurovascular Microemboli After Transcatheter Aortic Valve Replacement
Source: J Soc Cardiovasc Angiogr Interv. 2023 Nov 30;3(1):101180. doi: 10.1016/j.jscai.2023.101180 (PMC11308225; doi:10.1016/j.jscai.2023.101180)
Supplement: Supplemental Data [file mmc1.docx]

| **Supplementary Data: Wilcoxon Rank Sum Test Analysis of Continuous Variables** | |
| --- | --- |
| **Continuous Variable** | **p value** |
| Fluoroscopy time | 0.38 |
| sheath size | 0.20 |
| Age | 0.39 |
| Society of Thoracic Surgery Score | 0.67 |
| Hemoglobin | 0.34 |
| Hematocrit | 0.49 |
| INR | 0.24 |
| Creatinine | 0.40 |
| GFR | 0.46 |
| BMI | 0.65 |
| BSA | 0.35 |
| Hemglobin A1c | 0.88 |
| Ejection Fraction | 0.37 |
| AVA | 0.28 |
| AVAi | 0.85 |
| DOI | 0.89 |
| MPG | 0.45 |
| PV | 0.48 |
| SBP | 0.20 |
| SVI | 0.64 |
| E/E | 0.64 |
| Septal E | 0.09 |
| Lateral E | 0.38 |
| E/A | 0.55 |
| LAVi | 0.10 |
| ZVA | 0.37 |
| ∆ DOI | 0.48 |
| ∆ MPG | 0.67 |
| ∆ PV | 0.64 |
| ∆ SBP | 0.81 |
| ∆ SVI | 0.58 |
| ∆ E/E | 0.30 |
| ∆ Septal E | 0.12 |
| ∆ Lateral E | 0.06 |
| ∆ E/A | 0.67 |
| ∆ LAVi | 0.002 |
| ∆ ZVA | 0.37 |
| Total Calcium Score | 0.69 |
| Root Angle | 0.58 |
| Ascending Aorta Diameter | 0.29 |
| Systolic Annular Perimeter | 0.55 |
| Systolic Annular Area | 0.55 |
| Ascending Aorta Height | 0.85 |
| LDRS by BSA | 0.10 |

**∆** change in parameter; **AVA** – Aortic Valve Area; **AVAi** – Aortic Valve Area Index; **LAVi** – Left Atrial Volume Index; **MPG** – Mitral Pressure Gradient; **SBP** -- Systolic Blood Pressure; **SVI** – Stroke Volume Index; **ZVA** – Ventriculo-arterial Impedance, **BSA** – Body Surface Area; **LDRS** – Aortic Length diameter ratio in relation to sheath size
